# Successful treatment of cesarean scar pregnancy with transvaginal injection of absolute ethanol around the gestation sac via ultrasound

**DOI:** 10.1186/s12884-019-2468-3

**Published:** 2019-08-27

**Authors:** Fangfang Lu, Yuanming Liu, Wenjun Tang

**Affiliations:** 1 Department of Obstetrics and Gynecology, Affliated Hospital of Guilin Medical University, Guilin, 541000 Guangxi People’s Republic of China; 2 Department of Ultrasound, Affliated Hospital of Guilin Medical University, Guilin, 541000 Guangxi People’s Republic of China; 3 Department of Clinical Laboratory, Affliated Hospital of Guilin Medical University, Guilin, 541000 Guangxi People’s Republic of China

**Keywords:** Absolute ethanol, Cesarean scar pregnancy, Transvaginal ultrasonography

## Abstract

**Background:**

This study aims to evaluate the curative effect and complications in cesarean scar pregnancy (CSP) patients treated with a transvaginal injection of absolute ethanol (AE) around the gestation sac (GS) under ultrasound guidance.

**Methods:**

This retrospective clinical investigation analyzed 26 CSP patients treated at the Affiliated Hospital of Guilin Medical University in Guilin, Guangxi, China, between January 1, 2018 and January 30, 2019. Outcomes and complications were analyzed following treatment with AE.

**Results:**

Out of the entire group, 20 patients were successfully treated with a single AE injection, while the remaining six patients required two or three repeat injections. In 21 patients, the serum beta-human chorionic gonadotropin (β-hCG) level was reduced to > 50% 1 day after a single AE injection; in 19 patients, the serum β-hCG level was reduced to > 80% 4 days after a single AE injection. In all patients, the average time for serum β-hCG to reduce to normal levels (< 3.0 mIU/mL) was 36.50 ± 12.54 days. The overall cure rate of CSP by AE injection was 100%. Average length of hospitalization was 6.73 ± 3.66 days, with Patient 2 having the longest hospitalization at 17 days, and Patient 3 the shortest at 2 days. No adverse effects on hematopoietic, hepatic or renal function were observed in the short term.

**Conclusion:**

The study demonstrated that transvaginal injection of AE around the gestation sac under ultrasound guidance had good clinical effects, fewer complications, and merit as a novel treatment for CSP. However, larger multi-center trials are needed to confirm the safety and effectiveness of this treatment.

## Background

In recent years, the cesarean section (CS) rate has increased globally. With this rise, the incidence of cesarean scar pregnancy (CSP) has also increased, particularly in China. The reported morbidity of CSP ranges from 1/2216 to 1/1800 pregnancies, accounting for 4% of ectopic pregnancies [1, 2]. CSP, a long-term complication of CS, is defined as the implantation of the gestational sac at the uterine incision scar of the previous CS. If treatment of CSP is delayed, it may lead to several serious complications, including hemorrhage, uterine rupture, hysterectomy, and even loss of sequent fertility [3]. As such, standard management for CSP is timely termination of pregnancy.

Many treatments for CSP have been proposed. These include: uterine dilatation and curettage (D&C), hysteroscopy, laparoscopy, resection of CSP through a transvaginal approach, uterine artery embolization (UAE), high-intensity focused ultrasound, treatment by potassium chloride, treatment by systemic methotrexate (MTX), treatment by local MTX, and combined medical and surgical management [4–10]. The efficacy and safety of these CSP therapies have been assessed in many research studies. One systematic review of CSP treatment indicated the efficacy rate of systemic and/or local MTX was 62%, while surgical treatments were associated with a high success rate (≥96%) and low risk of hemorrhage (≤4%) [11]. Petersen et al. [10] systematically reviewed 2037 CSP patients and identified 14 different approaches, among which five were recommended for CSP treatment: transvaginal approach, hysteroscopy, laparoscopy, UAE in combination with D&C, and UAE in combination with D&C and hysteroscopy. Yamaguchi et al. [12] showed that transvaginal MTX injection cured eight CSP patients successfully. Another study reported the cure rate among 28 CSP patients at 100% via transvaginal ultrasound-guided embryo aspiration plus local MTX injection, an effective method with less complications or adverse effects [13]. However, the standard treatment protocol for CSP is not yet established.

Some data suggest absolute ethanol (AE) can also be used for ectopic pregnancy therapy. The first use of AE in the treatment of ectopic pregnancy was reported by Kaijima et al. [14] in 2006. Building on this study, Hisao et al. [15] recently reported a novel, less-invasive treatment for cervical pregnancy (CP) and CSP using local AE injection, which may be superior to MTX-based local injection therapy. It was shown that injection of AE into the lacunar space around the gestation sac rapidly decreased serum beta-human chorionic gonadotropin (β-hCG) [15]. The above research mainly studied the curative effect of AE injections on CP treatment. In our study, we focused on the treatment of CSP by transvaginal AE injection. The aim of the report was to evaluate the curative effect and complications among CSP patients treated with AE.

## Methods

Approved by the hospital’s ethics committee, this retrospective clinical study analyzed 26 CSP patients treated at the Affiliated Hospital of Guilin Medical University in Guilin, Guangxi, China, between January 1, 2018 and January 30, 2019. Patient data were collected through archived medical records and all patients were clearly informed of their treatment modalities, as well as the risk of pregnancy preservation. All patients provided signed consent prior to the intervention. Clinical characteristics such as age, gravida para, size of gestation sac (GS), uterine scar thickness, and fetal heartbeat were reviewed. The change in serum β-hCG level and size of GS were dynamically measured after treatment. The change in white blood cells (WBC), hemoglobin (Hb), blood platelets (PLT), alanine transaminase (ALT), aspartate transaminase (AST), creatinine (Cr), and blood urea nitrogen (BUN) were also analyzed before and two months after treatment. Patients for whom follow-up was not possible were excluded from the study.

### Diagnostic criteria of CSP

All patients became pregnant spontaneously. The diagnosis of CSP was based on standard sonographic [16, 17] findings, confirming the following: 1) No pregnancy sac in the uterine cavity or cervical canal; 2) The pregnancy sac was located in the scar of the previous cesarean section in the lower uterine segment; 3) Color doppler flow imaging showed high velocity and low obstruction of blood flow around the pregnancy sac; and 4) Continuity of the myometrium in the anterior uterine wall was interrupted, with the myometrium between the pregnancy sac and bladder wall thinner or even absent.

### Transvaginal injection of AE around the gestation sac

Administered without anesthesia, AE (Anhydrous Ethanol Injection; Xilong Scientific, Shantou, Guangdong, China) was injected in all patients around the GS using a 20-G puncture needle under guidance of high-intensity imaging transvaginal ultrasonography (TVU) (HD11XE, Philips, USA). In all patients, initial AE dose was between 4.0 and 15.0 mL (mean 8.38 ± 2.65 mL), depending on GS size and serum β-hCG level. When the GS was larger or level of serum β-hCG higher, the patient required a higher AE dose. Total AE dosage was between 4.0 and 30 mL (mean 11.15 ± 6.37 mL).

### Therapeutic evaluation

All patients were hospitalized for treatment. The effect of AE local injection was evaluated based on percentage decrease of serum β-hCG, calculated by dividing the initial level of serum β-hCG before the first AE injection. A second AE dose was given if the initial level of serum β-hCG was higher than 65000mIU/mL, or the serum β-hCG decrease was < 50% one day after local injection, or < 80% four days after local injection and the patient needed to be reassessed in subsequent days. For patients whose decline in β-hCG levels were not satisfactory after the second injection, additional doses were administered until the required decline in β-hCG was achieved. The serum β-hCG level was rechecked on the first day and fourth day after AE injection, followed by one week after AE injection, two or three days after one week, and at one month until the required level was reached (< 3.0 mIU/mL). Meanwhile, the size of the GS was dynamically measured by TVU after AE injection until it completely disappeared.

### Statistical analysis

SPSS 13.0 statistical software (IBM Corp., Armonk, NY, USA) was used to process all data. Descriptive statistics are given as standard deviation of the mean, frequency, and percentage. Paired sample t-tests were employed to assess the change of WBC, Hb, PLT, AST, ALT, Cr, and BUN before and after AE injections. A value of *P* < 0.05 was defined as statistically significant.

## Results

Table 1 shows the clinical features of the 26 CSP patients. Average patient age was 34.12 ± 5.39 years. Among the entire group, positive fetal heartbeat (FHB) was visible in 11 patients. In all patients, average uterine scar thickness was 4.07 ± 2.07 mm. A total of 20 patients were successfully treated with a single AE injection, with the remaining patients requiring two or three AE injections due to slowly decreasing serum β-hCG levels. Among the 26 patients, 18 received 4~10 mL of AE injection, five received 11~20 mL of AE injection, and three received 21~30 mL of AE injection. The overall cure rate of CSP by AE injection was 100%.

**Table 1:** The clinical features of 26 CSP patients treated with AE local injection

Patients	Age range (year)	GP	GA	CS	Initial β-hCG mIU/mL	GS mm	uterine scar thickness mm	FHB	β-hCG at 1 day after AE injection mIU/mL	β-hCG decrease rate(%)at 1 day	β-hCG at 4 day after AE injection mIU/mL	β-hCG decrease rate(%)at 4 day	time for β-hCG reduced to normal day	Initial volume of AE injection mL	Total volume of AE injection mL	No. of AE injections	hospital stay (day)
1	20–30	G2P1	6w1d	1	25,820	21 × 7	3.1	–	13,694	46.96	5820	77.46	44	5	15	3	14
2	41–50	G3P2	6w4d	2	84,663	28	3.0	+	41,344	51.17	17,938	78.81	40	9	17	2	17
3	41–50	G3P2	5w4d	1	2708	9 × 6	2.9	–	611	77.44	173.8	93.58	28	4	4	1	2
4	20–30	G3P2	9w6d	1	125.4	29 × 21	3.5	–	42.9	65.79	16.6	86.76	9	5	5	1	3
5	31–40	G4P1	8w0d	1	59,196	29 × 17	5.0	+	18,625	68.54	4738	92.00	40	9	9	1	7
6	31–40	G5P2	7w0d	2	63,052	37 × 10	2.9	–	26,222	58.41	4630	92.66	40	10	10	1	5
7	31–40	G5P1	8w2d	1	17,165	17	6.0	+	6279	63.42	1631	90.50	32	6	6	1	3
8	31–40	G2P1	7w5d	1	10,801	27 × 15	4.6	–	3307	69.38	425.9	96.06	20	5	5	1	6
9	20–30	G3P1	6w1d	1	13,466	18 × 12	3.0	+	4890	63.69	1234	90.84	43	7	7	1	6
10	20–30	G4P3	5w6d	3	42,061	19 × 13	2.0	+	20,432	51.42	12,112	71.20	30	6	16	2	15
11	31–40	G3P2	6w3d	2	14,827	16	2.4	–	6149	58.52	2048	86.19	36	5	5	1	4
12	31–40	G6P2	8w0d	2	36,177	18	3.0	–	20,785	42.55	5290	85.38	34	10	10	1	5
13	31–40	G2P1	6w5d	1	22,038	17	3.9	–	3111	85.88	2090	90.52	30	15	15	1	5
14	31–40	G9P2	7w2d	2	85,264	22 × 16	3.2	–	46,463	45.51	27,611	67.62	60	12	24	2	9
15	31–40	G3P1	9w4d	1	65,103	39 × 20	3.4	+	32,139	50.63	19,500	70.05	60	10	22	2	5
16	31–40	G10P2	6w4d	2	1203.4	23 × 14	1.4	–	491.4	59.15	190	84.21	29	6	6	1	3
17	20–30	G5P1	6w4d	1	37,002	26 × 10	4.9	+	16,056	55.61	2310	93.76	29	10	10	1	6
18	31–40	G4P1	6w6d	1	20,162	23 × 9	9.1	+	5012	68.36	931.5	95.38	25	8	8	1	6
19	31–40	G3P2	6w5d	2	93,544	24 × 12	4.0	+	61,432	34.33	49,768	46.80	60	10	30	3	9
20	20–30	G4P2	7w3d	2	56,255	32 × 20	5.9	+	26,486	52.92	5270	90.63	60	10	10	1	8
21	31–40	G3P2	5w5d	2	34,783	34 × 12	3.1	–	21,152	39.19	4312	87.60	40	8	8	1	6
22	20–30	G3P1	7w5d	1	46,520	31 × 17	3.0	–	22,870	50.84	2404	94.83	30	8	8	1	8
23	31–40	G3P1	7w2d	1	51,065	27 × 15	4.3	–	20,341	60.17	19,030	62.73	33	8	8	1	5
24	20–30	G4P1	8w0d	1	20,714	28 × 15	3.0	+	8216	60.34	927	95.52	31	10	10	1	6
25	41–50	G5P1	7w0d	1	3612	35 × 23	10.9	–	1222	66.17	432	88.04	31	12	12	1	7
26	31–40	G2P1	7w1d	1	19,316	16	4.2	–	5673	70.63	936	95.15	35	10	10	1	6

Abbreviations *AE* absolute ethanol; *GP* gravida para; *GA* gestational age; *CS* cesarean section; *β-hCG* beta-human chorionic gonadotropin; *GS* gestational sac; *FHB* fetal heartbeat; *CSP* cesarean scar pregnancy

In four patients (2, 14, 15, 19), the initial level of serum β-hCG was higher than 65000mIU/mL. Patients 2, 14, and 15 received second AE injections. Patient 19 received a third AE injection as the decline in β-hCG level was not obvious after the second injection(< 30%). In subsequent observation, all of them reached the required β-hCG level. In 21 CSP patients, serum β-hCG level was reduced > 50% one day after a single AE injection; this included Patients 2, 10 and 15, and Patient 13 who had the greatest decrease at 85.88%. Patient 10 received repeated injections due to serum β-hCG level reduction < 80% four days after a single AE injection; subsequently, the β-hCG level gradually decreased to normal (patient 2 and 15 have shown above). In another five patients (1, 12, 14, 19, 21), the decrease of serum β-hCG was < 50% and the smallest decline occurred in Patient 19 at 34.33%. Patient 1 received third injections due to the decline in β-hCG level not being obvious after the second injection (< 30%) and finally decreased to an acceptable level. Patient 12 and 21 only received a single AE injection as their serum β-hCG level reduction was > 80% 4 days after injection and the required β-hCG level was reached eventually (patients 14 and 19 as shown above). In 19 patients, the serum β-hCG level decreased > 80% 4 days after a single AE injection, while decreased < 80% in the other seven patients (1, 2, 10, 14, 15, 19 and 23). Patient 23 only received a single AE injection due to serum β-hCG level reduction > 50% 1 day after injection, then decreasing to an acceptable level (patients 1,2,10,14,15 and 19 has shown above). In all patients, average time for serum β-hCG to reach normal (< 3.0 mIU/mL) was 36.50 ± 12.54 days. Average length of hospitalization was 6.73 ± 3.66 days. Patient 2 had the longest hospitalization at 17 days, while Patient 3 had the shortest at 2 days.

**Fig. 1:**
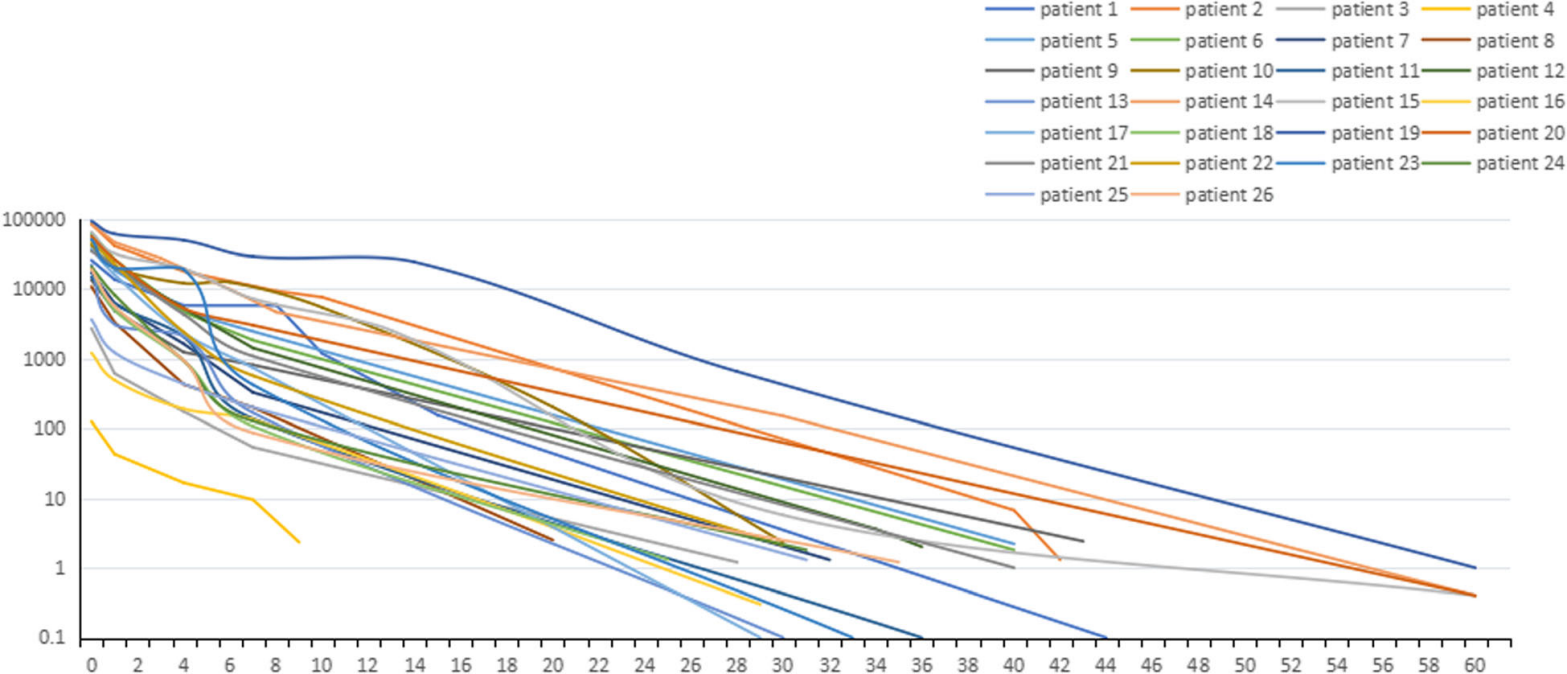
Decrease of serum β-hCG in CSP patients after AE injection. Days after treatment are plotted on the x-axis; decrease of serum β-hCG is plotted on the y-axis. In most patients, time for serum β-hCG to reduce to normal is 30 to 40 days. Patients 14, 15, 19, and 20 experienced the longest period, 60 days. Patient 4 had the shortest period, 9 days

**Fig. 2:**
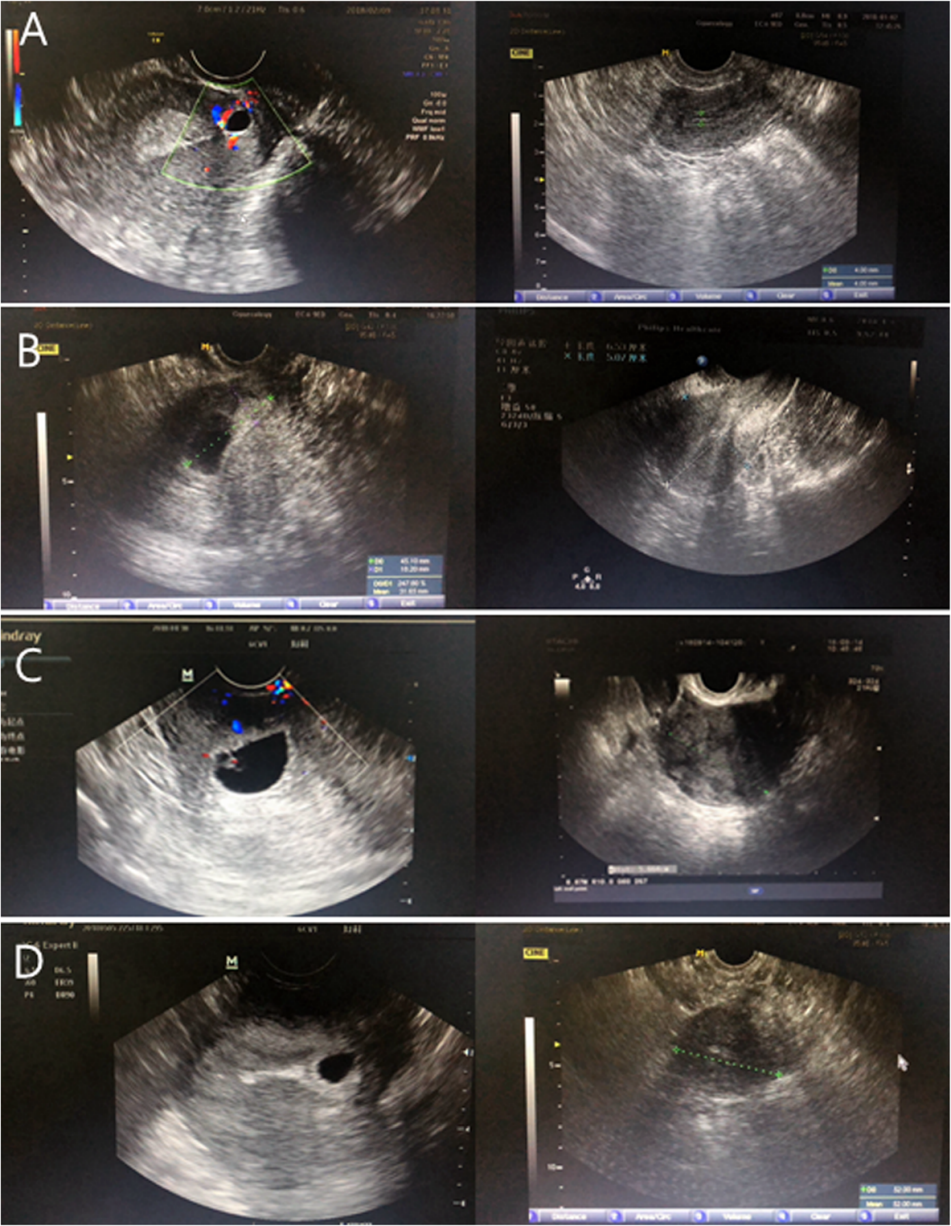
Change in TVU after AE injection for patients 3, 5, 7, and 11 (**a**) Change in TVU for Patient 3. **b** Change in TVU for Patient 5. **c** Change in TVU for Patient 7. **d** Change in TVU for Patient 11.

Decline of serum β-hCG levels in all CSP patients is shown in Fig. 1. In the of majority patients, time for serum β-hCG to drop to normal was 30 to 40 days. Patients 14, 15, 19, and 20 required the longest time (60 days) and Patient 4 the shortest (9 days). Figure 2 shows the change of TVU after AE injections for patients 3, 5, 7, and 11.

We further analyzed the change of WBC, Hb, PLT, ALT, AST, Cr, and BUN in all patients to evaluate short-term adverse effects of AE injection on hematopoietic function or hepatic and renal function. Table 2 shows values of Hb, WBC, and PLT before AE injection were about 127.00 ± 11.27 g/L, 8.61 ± 1.96×10^9/L, and 264.88 ± 58.80×10^9/L, respectively. These values were not significantly different from those 2 months after AE injection (*P* > 0 .05). Values of ALT and AST before AE injection were 15.33 ± 10.38 U/L and 14.62 ± 5.51 U/L, respectively; the difference was not statistically significant compared with values at 2 months after AE injection (*P* > 0.05). Values of Cr and BUN were also similar before and 2 months after AE injections, as shown in Table 2 (*P* > 0.05). One patient (Patient 2) required symptomatic treatment for persistent bleeding (> 500 mL) and a moderate hematoma (Hb 84 g/L).

**Table 2:** Comparison of changes in routine blood index, liver function, and renal function before and after AE injection

	Mean ± SD		*P* value
	Before injection	Two month after injection	
Hb(g/L)	127.00 ± 11.27	125.86 ± 13.77	0.626
WBC(×10^^9^/L)	8.61 ± 1.96	8.65 ± 1.70	0.901
PLT(×10^^9^/L)	264.88 ± 58.80	269.38 ± 63.94	0.603
ALT(U/L)	15.33 ± 10.38	14.93 ± 7.75	0.774
AST(U/L)	14.62 ± 5.51	15.30 ± 5.85	0.409
Cr (umol/L)	52.51 ± 8.44	50.23 ± 10.58	0.144
BUN (mmol/L)	4.21 ± 1.80	4.53 ± 1.45	0.063

## Discussion

CSP is a rare type of ectopic pregnancy, which has increased in recent years due to a rise cesarean sections. As CSP may cause serious complications – including hemorrhage, uterine rupture, and hysterectomy – early diagnosis and timely treatment are critical. Affecting women of reproductive age, most patients tend to choose conservative treatment for pregnancy termination, desiring to preserve the uterus and retain reproductive function. In any case, the best treatment for CSP remains unclear.

Currently, many conservative strategies have been established, including systemic and/or local MTX, D&C, hysteroscopy, laparoscopy, transvaginal resection, UAE, high-intensity focused ultrasound, and combined treatment [10, 18–21]. Among these, systemic and local MTX are the most widely used forms of management for CSP due to their minimally invasive nature [22]. MTX is a dihydrofolate reductase inhibitor used in the treatment of autoimmune diseases, malignancy, and as an abortifacient [1]. In CSP treatment, the mechanism of action of MTX is the inhibition of embryonic growth by causing the destruction of trophoblast cells and reducing local tissue blood flow, subsequently leading to embryonic death. Once trophoblast cells are destroyed, β-hCG is released into the blood, ultimately leading to an initial increase in serum β-hCG levels. Response to MTX occurs over 5 to 7 days [15]. Previous studies on MTX treatments provided conflicting results, likely due to differences in study design, definition of response, and additional treatment. Some researchers have confirmed the effectiveness of MTX and recommend it as the first choice for conservative treatment [12, 23]. Conversely, another review showed that nearly a quarter of patients treated with systemic MTX needed additional treatment, and severe complications occurred in 13% of cases [10]. Repeated use of MTX may result in genital infection, leukopenia, hepatic dysfunction, and vaginal bleeding [13]. For reproductive age women, MTX also showed embryo toxicity and teratogenicity. If treated with MTX, CSP patients require restrictive contraception for at least 3 months before a subsequent pregnancy. Therefore, it is necessary to explore more effective and less complicated CSP treatment methods.

As early as 1973, researchers reported the use of AE for the induction of mid-trimester abortions [24]. Subsequently, in 2006, AE injection was first used for ectopic pregnancy therapy [14]. Recently, Hisao et al. [15] reported usage of transvaginal AE injection for cervical pregnancy and CSP, known as “trophoblast target therapy” (TTT). They focused on 16 cervical pregnancy patients and 3 CSP patients, showing a successful outcome of TTT with AE injections. In our study, we used local injection of AE guided by transvaginal sonography to treat 26 CSP patients with gestational ages (GA) ranging from 5 weeks to 9 weeks and 6 days. We saw clinical success, with a curative rate of 100%. Notably, 20 patients (76.92%) underwent only one injection and attained a satisfactory result, while the remaining 6 patients (23.08%) required repeat AE injections due to serum β-hCG levels decreasing slowly. In all patients, average time for serum β-hCG to reduce to normal (< 3.0 mIU/mL) was 36.50 ± 12.54 days. The time for serum β-hCG levels to reduce to normal for the majority of patients ranged from 30 to 40 days. A few patients required 60 days, possibly related to high initial β-hCG levels or different sensitivity to AE treatment. This indicated that AE local injection around the gestation sac was effective for early pregnancy between 5 weeks to 9 weeks and 6 days, and also had an obvious effect on cases with positive FHB. However, it takes a long time for β-hCG to normalize. The mechanism of action for AE is causing trophoblastic necrosis through coagulation and dehydration, thus inhibiting proliferation of trophoblast cells, rapidly causing a reduction in serum β-hCG levels after initial injection. Furthermore, ethanol was metabolized quickly and did not accumulate in the body, making low-dose AE non-toxic, allowing for repeated injection as needed. Among 26 patients, 69.23% received 4~10 mL of AE injection, 19.23% received 11~20 mL of AE injection, and 11.54% received 21~30 mL of AE injection. Furthermore, we compared WBC, Hb, PLT, AST, ALT, Cr, and BUN in all patients before and at 2 months after AE injections. Our results demonstrated that there were no significant differences in hematopoietic function, liver function, and renal function after treatment, indicating AE injection has no adverse effects on hematopoietic function or hepatic and renal function in the short term.

Massive hemorrhage is a common complication of CSP. In this study, only Patient 2 reported blood loss in excess of 500 mL, subsequently requiring treatment. None of the patients experienced devastating hemorrhage. Uterine rupture is another serious complication. Li et al. [13] showed that the risk of uterine rupture is increased in patients having the gestational sac near the serous layer of the uterus. With expectant management, other researchers showed that the incidence of uterine rupture in CSP patients with embryonic/fetal heart activity was 9.9%, and hysterectomy was required in 15.2% during the first or second trimester, while uterine rupture rarely occurred in CSP patients without embryonic/fetal heart activity [25]. A recent review showed patients with multiple CS history and uterine scar thickness of < 2 mm were more likely to suffer from complications [26]. In all 26 patients, average cesarean scar thickness was 4.07 ± 2.07 mm. Patient 25 had the thickest uterine scar (10.90 mm) and Patient 16 had the thinnest uterine scar (1.40 mm). No uterine rupture was observed in any patient. It is notable that preserving the uterus is a major concern for both patients and their physicians, as it may influence menstruation and future fertility plans. In our study, all patients were treated successfully by AE local injection and did not require hysterectomy after treatment, thereby preserving their reproductive ability. Despite serious complications of CSP, some patients choose to continue pregnancy as reported in some research [25, 27]. Timor et al. [25] showed that a significant proportion of CSP patients who declined pregnancy termination progressed to the third trimester; thus, they considered termination of pregnancy as not the only therapeutic option offered to these patients. However, 39.2% of patients had severe bleeding, while uterine rupture occurred in 10.2% of patients, with most of them diagnosed as having abnormally invasive placenta at delivery. Therefore, in clinical practice, CSP patients should be fully aware of the risks of continuing pregnancy, enabling them to make informed decisions regarding termination. However, without follow-up studies, we cannot draw conclusions about long-term complications and reproductive function.

This report had some weaknesses, including its retrospective nature, and small number of CSP cases, possibly leaving the results open to bias. Due to the absence of comparison with other CSP treatments, we cannot fully assess if AE local injection is superior to other conservative treatments. Another limitation is that all patients in our study were not assessed with long-term follow-up, so other potential complications of AE and its effect on fertility are unknown.

## Conclusions

Transvaginal injection of AE around the gestation sac under the guidance of ultrasound showed good clinical effects and can be used as a novel treatment for CSP. CSP patients treated with AE local injections can attempt spontaneous pregnancy or in vitro fertilization and embryo transfer (IVF-ET) treatment without contraception. However, this retrospective clinical investigation involved a small sample size and may have some limitations. Future studies with larger multi-center trials are needed to confirm the safety and effectiveness of this treatment.

## Data Availability

We agree to allow the readers and journal to review our primary data if requested.

## Abbreviations

AE: Absolute ethanol
ALT: Alanine transaminase
AST: Aspartate transaminase
BUN: Blood urea nitrogen
CP: Cervical pregnancy
Cr: Creatinine
CS: Cesarean sections
CSP: Cesarean scar pregnancy
D&C: Dilation and curettage
FHB: Fetal heartbeat
GA: Gestational age
GP: Gravida para
Hb: Hemoglobin
MTX: Methotrexate
PLT: Blood platelets
TTT: Trophoblast target therapy
TVU: Transvaginal ultrasonography
UAE: Uterine artery embolization
WBC: White blood cells
β-hCG: Beta-human chorionic gonadotropin
